# Supplementary material for: Understanding disease-associated metabolic changes in human colonic epithelial cells using the iColonEpithelium metabolic reconstruction
Source: PLoS Comput Biol. 2025 Jul 3;21(7):e1013253. doi: 10.1371/journal.pcbi.1013253 (PMC12240309; doi:10.1371/journal.pcbi.1013253)

Independent Section

Contains tests that are independent of the class of modeled organism, a model's complexity or types of identifiers that are used to describe its components. Parameterization or initialization of the network is not required. See readme for more details.

Consistency

Test	Result
Stoichiometric Consistency	100.0% X3
Mass Balance	96.0%
Charge Balance	64.0%
Metabolite Connectivity	100.0%
Unbounded Flux In Default Medium	58.9%
Sub Total	88% X3

Annotation - Metabolites

Test	Result
Presence of Metabolite Annotation	96.6%
Metabolite Annotations Per Database	Info
pubchem.compound	0.0%
kegg.compound	35.0%
seed.compound	36.3%
inchikey	0.0%
inchi	0.1%
chebi	37.8%
hmdb	36.1%
reactome	0.0%
metanetx.chemical	70.4%
bigg.metabolite	96.5%
biocyc	34.4%
Metabolite Annotation Conformity Per Database	Info
pubchem.compound	100.0%
kegg.compound	100.0%
seed.compound	100.0%
inchikey	0.0%
inchi	100.0%
chebi	100.0%
hmdb	100.0%
reactome	0.0%
metanetx.chemical	100.0%
bigg.metabolite	100.0%
biocyc	100.0%
Uniform Metabolite Identifier Namespace	100.0%
Sub Total	77%

Annotation - Reactions

Test	Result
Presence of Reaction Annotation	97.9%
Reaction Annotations Per Database	Info
rhea	11.7%
kegg.reaction	9.7%
seed.reaction	16.7%
metanetx.reaction	55.5%
bigg.reaction	95.5%
reactome	0.0%
ec-code	13.1%
brenda	0.0%
biocyc	11.6%
Reaction Annotation Conformity Per Database	Info
rhea	99.9%
kegg.reaction	100.0%
seed.reaction	100.0%
metanetx.reaction	100.0%
bigg.reaction	100.0%
reactome	0.0%
ec-code	99.6%
brenda	0.0%
biocyc	100.0%
Uniform Reaction Identifier Namespace	100.0%
Sub Total	75%

Annotation - Genes

Test	Result
Presence of Gene Annotation	99.7%
Gene Annotations Per Database	Info
refseq	0.0%
uniprot	0.0%
ecogene	0.0%
kegg.genes	0.0%
ncbigi	10.5%
ncbigene	99.0%
ncbiprotein	0.0%
ccds	97.6%
hprd	52.9%
asap	0.0%
Gene Annotation Conformity Per Database	Info
refseq	0.0%
uniprot	0.0%
ecogene	0.0%
kegg.genes	0.0%
ncbigi	89.5%
ncbigene	100.0%
ncbiprotein	0.0%
ccds	100.0%
hprd	100.0%
asap	0.0%

Specific Section

Covers general statistics and specific aspects of a metabolic network that are not universally applicable. See readme for more details.

SBML

Test	Result
SBML Level and Version	SBML Level 3 Version 1
FBC enabled	true

Basic Information

Test	Result
Model Identifier	iColonEpithelium
Total Metabolites	4,068
Total Reactions	6,651
Total Genes	1,960
Total Compartments	10
Metabolic Coverage	3.39
Unconserved Metabolites	0
Minimal Inconsistent Net Stoichiometries	0

Metabolite Information

Test	Result
Unique Metabolites	2,027
Duplicate Metabolites in Identical Compartments	0
Metabolites without Charge	0
Metabolites without Formula	0
Medium Components	852

Reaction Information

Test	Result
Purely Metabolic Reactions	3,140
Purely Metabolic Reactions with Constraints	21
Transport Reactions	2,504
Transport Reactions with Constraints	10
Reactions With Partially Identical Annotations	0.19
Duplicate Reactions	0.00
Reactions With Identical Genes	0.52

Gene-Protein-Reaction (GPR) Associations

Test	Result
Reactions without GPR	1,519
Fraction of Transport Reactions without GPR	0.47
Enzyme Complexes	Errored

Biomass

Test	Result
Biomass Reactions Identified	1
Biomass Consistency	Errored
Biomass Production In Default Medium	204.99
Unrealistic Growth Rate In Default Medium	true
Biomass Production In Complete Medium	552.33
Blocked Biomass Precursors In Default Medium	0
Blocked Biomass Precursors In Complete Medium	0
Ratio of Direct Metabolites in Biomass Reaction	0.09
Number of Missing Essential Biomass Precursors	13

Energy Metabolism

Test	Result
Non-Growth Associated Maintenance Reaction	Errored
Growth-associated Maintenance in Biomass Reaction	false
Number of Reversible Oxygen-Containing Reactions	12
Erroneous Energy-generating Cycles	Info
MNXM3	Errored
MNXM63	Errored
MNXM51	Errored
MNXM121	Errored
MNXM423	Errored
MNXM6	0
MNXM10	0
MNXM38	0
MNXM208	Skipped
MNXM191	Skipped
MNXM223	Skipped
MNXM7517	Skipped
MNXM12233	Skipped
MNXM558	Skipped
MNXM21	Errored
MNXM89557	Errored

Network Topology

Test	Result
Universally Blocked Reactions	0
Orphan Metabolites	0
Dead-end Metabolites	0
Stoichiometrically Balanced Cycles	1,359
Metabolite Production In Complete Medium	335
Metabolite Consumption In Complete Medium	335

Matrix Conditioning

Test	Result
Ratio Min/Max Non-Zero Coefficients	0.00
Independent Conservation Relations	131
Rank	3937
Degrees Of Freedom	2714

Experimental Data Comparison

Test	Result
Growth Prediction	Skipped
Gene Essentiality Prediction	Skipped

Misc. Tests

Environment

Python Version	3.10.13
Platform	Linux
Memote Version	

Annotation - SBO Terms

Metabolite General SBO Presence	96.5%
Metabolite SBO:0000247 Presence	96.5%
Reaction General SBO Presence	97.8%
Metabolic Reaction SBO:0000176 Presence	99.7%
Transport Reaction SBO:0000185 Presence	94.4%
Exchange Reaction SBO:0000627 Presence	99.9%
Demand Reaction SBO:0000628 Presence	100.0%
Sink Reactions SBO:0000632 Presence	100.0%
Gene General SBO Presence	99.7%
Gene SBO:0000243 Presence	99.7%
Biomass Reactions SBO:0000629 Presence	100.0%
Sub Total	99% X2
Total Score	89%

Total Score

89%

Score per Category
Export

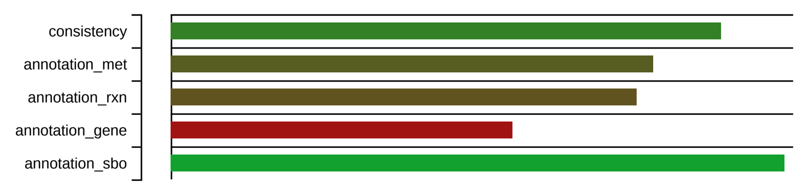

Supplement: S2 Data — (PDF) [file pcbi.1013253.s002.pdf]
