# Supplementary figures and images for: Understanding disease-associated metabolic changes in human colonic epithelial cells using the iColonEpithelium metabolic reconstruction
Source: PLoS Comput Biol. 2025 Jul 3;21(7):e1013253. doi: 10.1371/journal.pcbi.1013253 (PMC12240309; doi:10.1371/journal.pcbi.1013253)

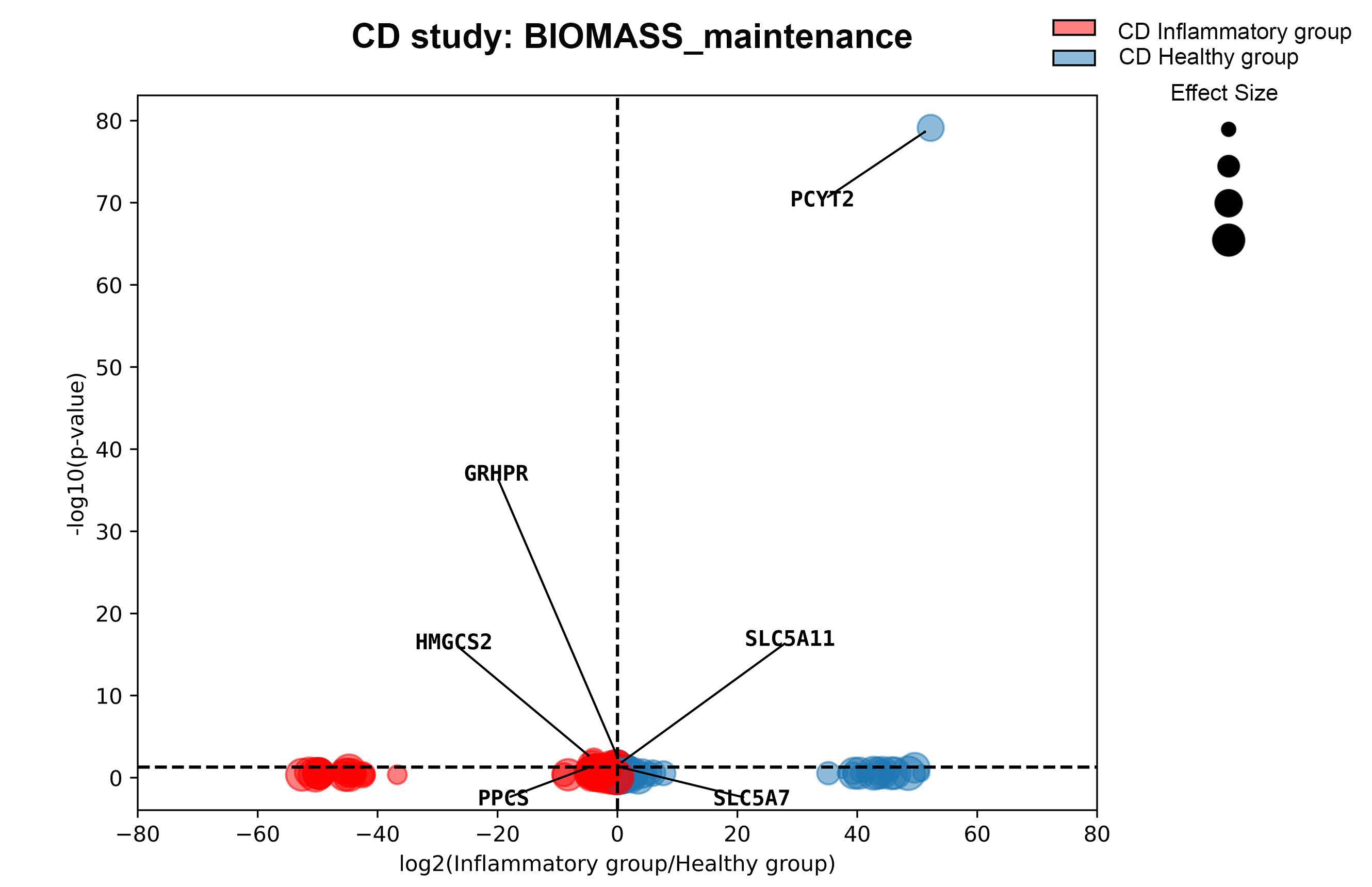

Supplement: S1 Fig — (TIFF) [file pcbi.1013253.s004.tiff]
